# Clinical Models of Care for Adults With Intellectual Disabilities in Forensic Mental Health Services: A Scoping Review

**DOI:** 10.1111/jir.70048

**Published:** 2025-10-13

**Authors:** Alina Haines‐Delmont, Dineesha Georgeena Rajan, Sian Cooper, Faye McLoughlin, Sahrish Ali, Katie Goodall, Joy Duxbury, Faith Hurley, Camilla Lindekilde, Michaela Thomson, Rachel Whyte, Erica Hateley, Tella Lantta

**Affiliations:** ^1^ School of Nursing and Public Health Manchester Metropolitan University Manchester UK; ^2^ Greater Manchester Mental Health Foundation NHS Trust Manchester UK; ^3^ Northern Care Alliance NHS Foundation Trust Manchester UK; ^4^ Mersey Care NHS Foundation Trust Prescot UK; ^5^ Institute of Health, University of Cumbria Lancaster UK; ^6^ Forensic Mental Health Research Unit Middelfart, Department of Regional Health Research, Faculty of Health Science University of Southern Denmark Middelfart Denmark; ^7^ Psychiatric Department Middelfart Mental Health Services in the Region of Southern Denmark Middelfart Denmark; ^8^ Institute of Psychiatry, Psychology and Neuroscience King's College London London UK; ^9^ Latrobe City Council Churchill Victoria Australia; ^10^ Department of Nursing Science, Faculty of Medicine University of Turku Turku Finland; ^11^ Centre for Forensic Behavioural Sciences Swinburne University of Technology Melbourne Australia

**Keywords:** forensic psychiatry, intellectual disabilities, mental health services, organisational models, patient care planning, patient‐Centred care

## Abstract

**Background:**

People with intellectual disabilities (ID) and forensic histories face significant health inequalities, including reduced quality of life and prolonged stays in mental health hospitals. This is a global health issue, and there is an urgent need for evidence‐based specific forensic interventions, models of care and service models to allow for effective discharge in the community, improve long‐term outcomes and reduce healthcare costs.

**Method:**

This scoping review was conducted in accordance with the Preferred Reporting Items for Systematic Reviews and Meta‐Analyses (PRISMA) Extension for Scoping Reviews. We have adapted Morrisey's framework to report outcomes of clinical models of care to include (i) effectiveness of treatment; (ii) patient safety; (iii) patient and family experience of care; and (iv) staff outcomes, skills and attributes.

**Results:**

Fifty‐six studies were included in this review, reporting on 49 interventions, models of care and service models (referred to as ‘models’). Four forensic models of care were identified as best practice: the Discharge Pathway Protocol, the Care Pathway‐Based Approach, the Psychological Treatment Pathway and the Forensic Intellectual Disability Secure Services (FIDSS) Model of Care. The first three have demonstrated effectiveness in reducing length of stay, facilitating timely discharges and improving patient outcomes for individuals with ID, while the FIDSS Model of Care represents a holistic and culturally sensitive approach emphasising person‐centred care, rehabilitation and quality of life. The findings underscore the need for larger studies to explore predictors of successful discharge and long‐term outcomes.

**Conclusions:**

This is the first review to bring together ‘clinical effectiveness’ studies and those reporting on patient and family experience, as well as staff's needs, attributes and experiences. Policymakers and practitioners should consider the models identified here as frameworks for developing effective, person‐centred care pathways, ensuring appropriate staff training and support, meaningful communication and work with the patient and their family/peers/support network and integrating community services to address the complex needs of this vulnerable population.

## 1 Background

People with intellectual disabilities face significant health inequalities when it comes to access to health care, quality of life and life expectancy (White et al. 2023). Intellectual disabilities (ID)—also referred to as learning disabilities (LD)—are irreversible lifelong conditions that usually start before adulthood and comprise significant global intellectual impairment with a functional IQ of < 70 and impairment of adaptive behaviour (Brown et al. 2010). It is well established that people with ID and additional forensic history often stay in secure hospitals for many years, sometimes decades (Fazel et al. 2016; Völlm et al. 2017). Criticism regarding forensic treatment for people with ID has led to a policy drive (in the United Kingdom and internationally) to prioritise discharge into appropriate community placements (Department of Health and Social Care 2024), but specific guidance is lacking (NICE 2016). It is important that, in the drive toward community treatment options, avoidance of hospitalisation and adaptations of Mental Health legislation, this forensic cohort does not become a forgotten population. Analysis of care pathways reveals a risk of multiple transfers between levels of security, with decisions often made in a reactive manner. There are multiple factors that contribute to such pathway management choices; however, this represents a health inequality and indeed one that has pervasive and life‐limiting consequences (Taylor et al. 2017). The human costs of getting it wrong are immense, not only for the individual but also for other vulnerable people within society (Beadle‐Brown et al. 2010; Esan et al. 2015). In addition, the health expenditure in this sector is significantly high, with an estimated 300 million pounds sterling per annum in the United Kingdom (Alexander et al. 2011).

Given these significant and timely issues, it is important to develop a model of care that is better suited to ensure prospective care planning and an appropriate and accelerated discharge pathway for better outcomes in the long term (NHS England 2023). This scoping review is part of a larger UK study commissioned by the National Health Service (NHS) (IRAS ID 324747) and it aims to synthetise the evidence‐base regarding models of care to improve practice and outcomes for this cohort of people who have been shown to experience significant health inequalities.

While systematic reviews have examined specific interventions for people with intellectual disabilities in forensic settings (e.g., psychological therapies and offender treatment programmes [Morrissey et al. 2017]), there has been no comprehensive synthesis of interventions, models of care or service models as a whole. Existing reviews tend to focus on single intervention types or narrow outcome domains, rather than mapping the range of available models, their components and the evidence supporting them. This absence of a consolidated evidence base limits the ability of commissioners, service providers and clinicians to identify best practice approaches and align service delivery with proven or promising models. This scoping review addresses this gap by systematically mapping and describing service models, models of care or interventions part of models of care/service models reported in the international literature for forensic services for people with intellectual disabilities, and by identifying evidence‐based best practice. More specifically, the review's focus on models of care is practice‐improvement oriented, aiming to inform clinicians about appropriate and effective frameworks they can use to deliver treatments and manage patient care in forensic ID settings.

There is no consistent definition of a ‘model of care’ (MOC), and the term is used variably across health and social care literature, generally referring to an overarching framework that defines how services are delivered (implemented and evaluated) to meet the needs of a specific population (Davidson et al. 2006). While the characteristics of different models are unclear, MOCs typically describe the guiding principles, care components and organisational arrangements underpinning service delivery, rather than discrete therapeutic interventions. It includes processes like screening, assessment, diagnosis, treatment planning, delivery, review and discharge/referral—typically covering the full patient journey and focusing on person‐centred, evidence‐informed practice (Kennedy 2022). As noted by Kennedy (2022), the term is contested, with overlap and inconsistency in its use alongside related concepts such as ‘service models’, ‘care pathways’ and, in some contexts, specific interventions. Forensic intellectual disability (ID) literature often applies these terms interchangeably, which can obscure the boundaries between them.

For the purposes of this review, we adopted an inclusive definition of MOCs, including studies reporting on (i) explicitly labelled ‘models of care’ as well as those describing (ii) ‘service models’ (i.e., the structure, configuration and operational processes of a service in a specific context; usually translating the principles of an MOC into practical delivery mechanisms) or (iii) a single named ‘intervention’ (i.e., a specific, discrete clinical, psychological or rehabilitative programme or activity, designed to achieve particular outcomes for service users; usually delivered within, and supported by, an MOC or service model) (Davidson et al. 2006).

This decision reflects the heterogeneity and inconsistent terminology used in the literature, particularly in forensic ID services, where interventions are frequently embedded as central elements of a service's model of care. Grouping these under the broader construct of MOCs enabled a more comprehensive synthesis of the available evidence while retaining sensitivity to the conceptual distinctions between them.

## 2 Objective

This scoping review aims to answer the following primary question:

1 What clinical models of care, interventions and/or service models (‘models’) are utilised for people with ID in forensic mental health services?

Secondary research questions include the following:

2 What is known about these models' potential impact on patient outcomes, for example, admissions, length of stay, discharge and improvement in symptoms?
3 What is known about these models' potential impact on safety, for example, self‐harm, violence and the use of restrictive practices?
4 What is known about these models' potential impact on outcomes, attributes and needs of staff delivering care to people with ID in forensic mental health services?
5 What is known about patients and family members' expectations and experiences of care in forensic mental health services?

## 3 Methods

### 3.1 Study Design and Rationale

This review followed the Joanna Briggs Institute (JBI) methodology for scoping reviews (Peters et al. 2020) and the PRISMA‐ScR reporting guidelines (Tricco et al. 2018). A scoping review was chosen over other review types because our aim was to map the breadth and nature of the available evidence on models of care for people with intellectual disabilities in forensic mental health services, to clarify conceptual boundaries and to identify gaps in knowledge. The literature in this area is heterogeneous in terminology, study design and outcomes, making a scoping approach more appropriate than a systematic review focused solely on effectiveness (Arksey and O'Malley 2005; Levac et al. 2010).

**FIGURE 1:**
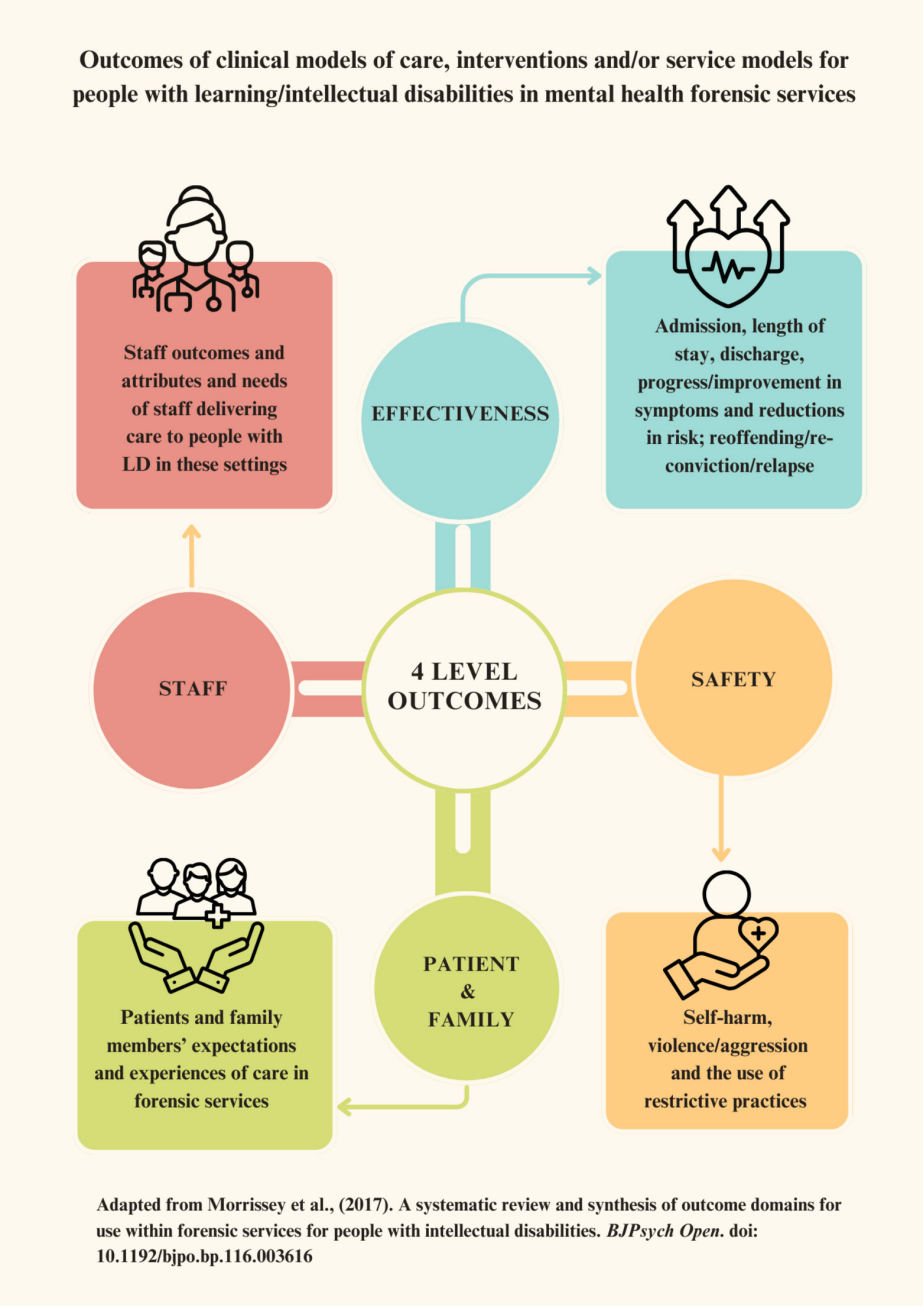
Adapted framework for capturing outcomes of clinical models of care for adults with learning/intellectual disabilities in forensic mental health services.

The protocol was registered on Open Science Framework ahead of conducting the searches (https://osf.io/5gqwc). The review team included experienced academics and practitioners in the area of intellectual disabilities, forensic mental health and coercive practices. We adapted Morrissey et al.'s (2017) framework for identifying and reporting on outcomes of models to cover: (i) effectiveness of treatment; (ii) patient safety; (iii) patient and family experience of care; and (iv) staff experiences, skills and attributes (Figure 1). Morrissey's framework was chosen as it provides a robust, structured method for categorising outcomes in forensic mental health service evaluations, encompassing three a priori superordinate domains: (a) effectiveness, (b) patient safety and (c) patient and carer experience. However, evidence from both ID and broader health service research indicates that staff‐related outcomes, such as skills, attributes, retention and well‐being are critical determinants of service quality and sustainability (Donabedian 2005; NHS England 2016). Given the relational and specialist nature of care in forensic ID services, staff competencies and experiences directly influence therapeutic alliance, patient outcomes and ward safety. To capture these factors systematically, we added a fourth superordinate domain: ‘staff outcomes, skills and attributes’. This adaptation allowed us to code and synthesise data on workforce elements that were prominent in the included studies but would otherwise have been excluded or subsumed, thereby enhancing the ecological validity and practical utility of the review's findings.

#### 3.1.1 Searching

We searched four electronic databases: CINAHL (EBSCO), Medline (Ovid), PsycINFO (EBSCO) and PubMed (NCB). The search strategies were adapted for different databases (see Supporting Information). All databases were searched from inception up to 31/08/2024.

#### 3.1.2 Inclusion and Exclusion Criteria

We included peer‐reviewed papers of any design published in the English language reporting on interventions, service models or models of care and associated outcomes for adults with a diagnosis of ID in forensic mental health services; comorbidities were included only if an ID diagnosis was present. See Table 1 for detailed inclusion/exclusion criteria and Table 2 for search strings.

**TABLE 1:** Inclusion/exclusion criteria.

	Inclusion	Exclusion
Population	Adults* (16+) of any gender or race with a diagnosis of ID (as defined by the authors), with possible comorbidities, for example, emotionally unstable personality disorder, anti‐social personality disorder, severe mental illness, autism. Adults (as above) under the care of forensic mental health services, including prisoners who require treatment within mental health settings	Children, people without intellectual disabilities diagnosis
Setting	Forensic mental health services, including prisoners who require treatment within mental health settings	Services exclusively for children/adolescent or elderly/older people populations or non‐ID populations
Interventions	Papers reporting on all types of interventions/models designed for people with ID, capturing a wide range of outcomes	Studies focusing on pharmacological interventions
Study type	Peer reviewed papers of any design published in the English language since 2000, as well as grey literature such as reports, guidelines, evaluations, audits and service descriptions.	Studies published in non‐English language and/or before 2000 Reviews, dissertations/theses, research protocols
Outcomes	Patients and family members' experiences/views; Staff experiences/views Patients and staff safety (use of restrictive practices; behaviour that challenges; violence and aggression; injury) Discharge into the community and/or transition to other services Staff training Individualised care plans Length of stay/admissions Quality of life.	

**TABLE 2:** Full search string as applied to Medline, Psychinfo, PubMed and CINAHL.

**Ovid MEDLINE(R)** ALL <1946 to August 23, 2024> Search run: 26/08/2024	1 exp intellectual disability/108036 2 exp learning disabilities/24381 3 exp autism spectrum disorder/45972 4 exp personality disorder/46140 5 exp developmental disabilities/22935 6 (learning disabilit* or learning disorder* or Ld or autis* or asd or autism spectrum disorder or asperger* or “developmental dis*” or “neurodevelopmental disorder” or “mental retard*” or “mental handicap*” or “mental impair*” or “mental subnormal*” or “mental deficiency” or “pervasive developmental disorder” or PDD or “developmental delay” or “challenging behavio?r*” or “behavio?r* of concern” or “behavio?r* that challenge*” or “aggressive behavio?r*”).mp. 242 365 7 or/1–6 366 383 8 (hospital* or unit* or facilit* or service* or inpatient or ward* or cent* or department* or clinic* organi#ation* or institution* or “assessment unit*” or forensic or secure or Specialist or “locked ward”).mp. 8 151 740 9 (“model* of care” or treat* or interven* or therap* or program* or outcome* or effect* or “patient admission” or Intake or re‐admission or discharg* or pathway*).mp. 20 066 058 10 7 and 8 and 9 57 217 11 limit 10 to dt = 20 230 411–20 241 231 4334
**APA PsycInfo** <1806 to August 2024 Week 4> Search run: 26/08/2024	1 exp. learning disabilities/or exp. learning disorders/37029 2 (learning disabilit* or learning disorder* or LD or autis* or asd or autism spectrum disorder or asperger* or “developmental dis*” or “neurodevelopmental disorder” or “mental retard*” or “mental handicap*” or “mental impair*” or “mental subnormal*” or “mental deficiency” or “pervasive developmental disorder” or PDD or “developmental delay” or “challenging behavio?r*” or “behavio?r* of concern” or “behavio?r* that challenge*” or “aggressive behavio?r*”).mp. 210 846 3 1 or 2 221 291 4 (hospital* or unit* or facilit* or service* or inpatient or ward* or cent* or department* or clinic* organi#ation* or institution* or “assessment unit*” or forensic or secure or Specialist or “locked ward”).mp. 1 678 243 5 (“model* of care” or treat* or interven* or therap* or program* or outcome* or effect* or “patient admission” or Intake or re‐admission or discharg* or pathway*).mp. 3 077 928 6 3 and 4 and 5 41 075 7 limit 6 to up = 20 230 411–20 241 231 2645
**PubMed** **S**earch run: 31/08/2024	1 Learning disabilities [MESH Major Topic] OR autis*[tw] OR asd[tw] OR autism spectrum disorder[tw] OR asperger*[tw] OR developmental disorder[tw] OR neurodevelopmental disorder[tw] OR mental retard[tw] OR mental handicap[tw] OR mental impair[tw] OR mental subnormal[tw] OR mental deficiency[tw] OR pervasive developmental disorder[tw] OR PDD[tw] OR developmental delay[tw] OR challenging behaviour[tw] OR aggressive behaviour[tw] OR mental illness[tw] OR mental health[tw] 460 350 2 hospital[tw] OR unit[tw] OR facilit[tw] OR service[tw] OR inpatient[tw] OR ward[tw] OR centre[tw] OR center[tw] OR department[tw] OR clinic[tw] OR[tw] ORganisation[tw] OR[tw] Organisation[tw] OR institution[tw] OR assessment[tw] OR forensic[tw] OR secure[tw] OR Specialist[tw] OR locked ward[tw] 2 149 445 3 model[tw] OR care[tw] OR treat[tw] OR intervention[tw] OR therapy[tw] OR therapies[tw] OR program[tw] OR outcome[tw] OR effect[tw] OR patient admission[tw] OR Intake[tw] OR re‐admission[tw] OR discharge[tw] OR pathway[tw] 15 632 865 4 #1 AND #2 AND #3 48 740 5 #4 AND 2023/04/11:2024/12/31[dp] 5270
**CINAHL Ultimate (EBSCO)** Search run: 31/08/2024	S1 “intellectual disabilit*” OR id OR SU learning disorders OR learning disorder* OR LD OR autis* OR asd OR autism spectrum disorder OR asperger* OR “developmental dis*” OR “neurodevelopmental disorder” OR “mental retard*” OR “mental handicap*” OR “mental impair*” OR “mental subnormal*” OR “mental deficiency” OR “pervasive developmental disorder” OR PDD OR “developmental delay” OR “challenging behavio#r*” OR “behavio#r* of concern” OR “behavio#r* that challenge*” OR “aggressive behavio#r*” 132 196 S2 hospital* OR unit* OR facilit* OR service* OR inpatient OR ward* OR cent* OR department* OR clinic* organi#ation* OR institution* OR “assessment unit*” OR forensic OR secure OR Specialist OR “locked ward” 2,718,692 S3 “model of care” OR treat* OR interven* OR therap* OR program* OR outcome* OR effect* OR “patient admission” OR Intake OR re‐admission OR discharg* OR pathway* 4 446 442 S4 S1 AND S2 AND S3 29 512 S5 EM 20230411–20 241 231 346 391 S6 S4 AND S5 2500

#### 3.1.3 Screening

We used the review platform COVIDENCE (http://www.covidence.org) to upload search results, screen articles and remove duplicates. Each paper was independently screened by one reviewer at the title and abstract screening stage and by two reviewers at the full text screening stage. Any conflicts were resolved by discussing with a third reviewer or with the review team.

#### 3.1.4 Data Extraction

Each paper was extracted by one reviewer and checked by a second reviewer using a data extraction form in COVIDENCE. Data extraction was informed by the research questions and included domains covering the specific details, including study identifiers (authors, date of publication, country of origin), study design and method, population and setting (including sample size, age, gender and diagnosis), concept (description of model), outcomes (including data collection tool and type of outcome) and results. The data extraction form was designed and piloted collaboratively by the review team prior to extraction.

#### 3.1.5 Data Synthesis

Data extracted within COVIDENCE was downloaded as an Excel spreadsheet, cleaned and synthesised narratively by TL, DR, AHD, SA and FM (with feedback from the wider team).

Extracted data were collated and synthesised narratively, with findings organised according to an adapted version of Morrissey's framework (see Figure 1). A qualitative content analysis approach (Elo and Kyngäs 2008) was used to systematically code and categorise data from included studies into predetermined and emergent categories. This allowed us to identify, group and interpret patterns in the characteristics, outcomes and contexts of the models described, in line with the review's objectives.

## 4 Findings

**FIGURE 2:**
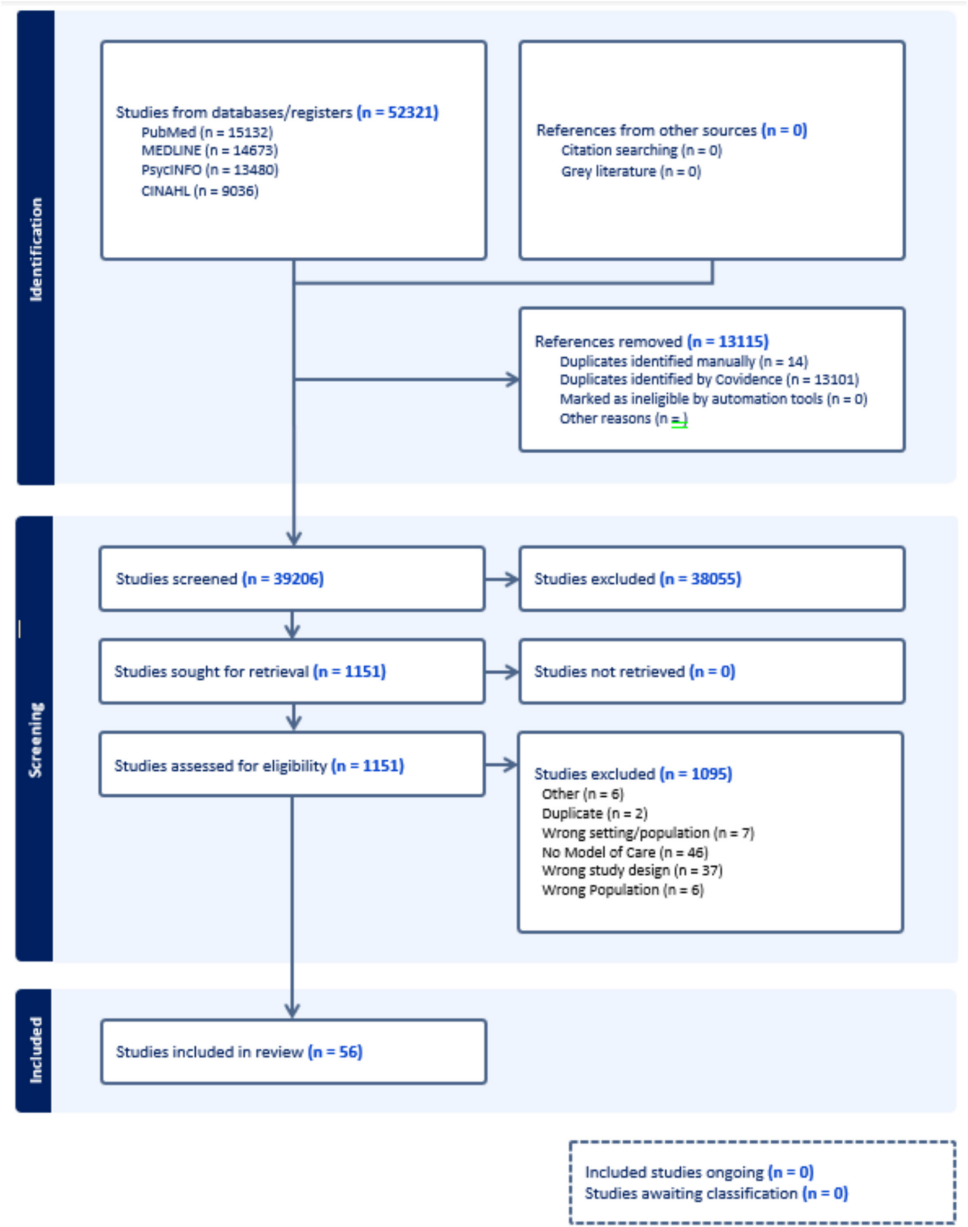
Prisma flow diagram (2020): Clinical Models of Care for Adults with Intellectual Disabilities in Forensic Mental Health Services.

The search generated 52,321 references. After application of the inclusion and exclusion criteria at two screening stages, 56 studies were included in this review (see Figure 2).

### 4.1 Characteristics of Included Studies (See Table 3)

**TABLE 3:** Key characteristics of included studies.

					Gender distribution								
Author	Year	Country	Research type	Sample size	M	F	Gender type	Setting	Population	Mean age (Age range)			Diagnosis
Alexander et al.	2011	UK (England)	Quantitative	138	138	0	Male	Inpatient	Patients	30.43 [NR]			ID with comorbid MH
Ashworth & Mooney	2016	UK (England)	Quantitative	35	35	0	Male	Inpatient	Patients	31.92 [18–55]			ID with comorbid MH
Ashworth et al.	2016	UK	Mixed methods	11	10	1	Mixed	Inpatient	Staff	NR			NR
Ashworth et al.	2017	UK	Mixed methods	NR	NR	NR	NR	Inpatient	Patients	NR			ID with comorbid MH
Ashworth et al.	2020	UK (England)	Quantitative	1	0	1	Female	Inpatient	Patients	23 [NR]			ID with neurodiversity
Ashworth et al.	2021	UK	Quantitative	5	5	0	Male	Inpatient	Patients	NR [23–57]			ID with comorbid neurodivergence & MH
Borghino et al.	2022	UK (England)	Qualitative	1	1	0	Male	Inpatient	Patients	35 [NR]			ID, ASC and Paedophilia
Browne et al.	2019	UK	Qualitative	9	4	5	Mixed	Inpatient	Patients	30.3 (9.03) [21–48]			ID
Browning et al.	2016	UK (England)	Mixed methods	70	66	4	Mixed	Community	Patients	37.1 [18–60]			ID
Campbell & McCue	2013	UK	Quantitative	5	2	3	Mixed	Inpatient	Patients	NR [34–68]			ID with comorbid MH
Chaplin et al.	2023	UK	Mixed methods	7	NR	NR	NR	Community	Staff	NR			ID with neurodevelopment
Chaplin et al.	2011	UK (England)	Qualitative	NR	NR	NR	Mixed	Community	Patients	[≥ 18]			ID with comorbid MH
Chaplin et al.	2021	UK	Quantitative	1281	NR	NR	Mixed	Criminal Justice	Patients	NR [18–76]			ID with neurodiversity
Cheshire et al.	2015	UK	Quantitative	63	23	40	Mixed	Inpatient	Patients	NR			ID
Chester et al.	2018	UK	Mixed methods	80	46	34	Mixed	Inpatient	Patients	NR			ID with TBI
Chester et al.	2015	UK	Qualitative	7	NR	NR	NR	Inpatient	Staff	NR			ID
Craven & Shelton	2020	UK	Quantitative	5	5	0	Male	Inpatient	Patients	33.8 (16) [20–61]			ID with comorbid MH
de Villiers & Doyle	2015	UK	Quantitative	117	114	3	Mixed	Community	Patients	30 [16–79]			ID
de Villiers & Johnstone	2024	UK (Scotland & Northern Ireland)	Quantitative	35	NR	NR	Mixed	Inpatient	Patients	NR			LD with comorbid MH and challenging behaviour
Delforterie et al.	2023	The Netherlands	Quantitative	250	235	14	Mixed	Inpatient	Patients	36.4 (11) [18.6–70.6]			ID with comorbid MH
Devapriam et al.	2014	UK	Quantitative	24	NR	NR	NR	Inpatient	Patients	[≥ 18]			ID with comorbid MH
Duff et al.	2023	New Zealand	Qualitative	NR	NR	NR	NR	Inpatient	Patients	NR			NR
Green & Cappleman	2023	UK (Wales)	Quantitative	13	NR	NR	NR	Inpatient	Staff	NR			ID with comorbid MH, Neurodiversity and challenging behaviour
Hackett et al.	2020	UK	Mixed methods	20	18	2	Mixed	Inpatient	Patients	33.2 [18+]			ID
Hickman et al.	2018a	UK	Qualitative	6	6	0	Male	Inpatient	Patients	NR			ID
Hickman & Morris	2022	UK (England)	Quantitative	16	16	0	Male	Inpatient	Patients	31 [21–60]			ID
Hickman et al.	2017	UK	Quantitative	28	28	0	Male	Inpatient	Patients	31.7 [19–57]			ID
Hickman et al.	2018b	UK	Quantitative	2	2	0	Male	Inpatient	Patients	NR [27–42]			ID
Inett et.al	2014	UK	Quantitative	28	28	0	Male	Inpatient	Patients	39 [18+]			ID with comorbid MH
Isherwood et al.	2006	UK	Qualitative	13	NR	NR	NR	Inpatient	Staff	NR			NR
Langdon et al.	2013	UK	Quantitative	7	7	0	Male	Inpatient	Patients	NR [21–36]			ID
Lindsay et al.	2013	UK (Scotland)	Quantitative	309	282	27	Mixed	Community	Patients	NR	ID	Lindsay et al.	2013
Lindsay et al.	2006	UK	Quantitative	247	226	21	Mixed	Community	Patients	SO	OO	F	ID
										36.3 [18–54]	31.6 [18–54]	28.8 [18–54]	
Lindsay et.al	2010	UK	Quantitative	197	NR	NR	Mixed	Community	Patients	18+			ID
McKinnon et al.	2024	UK (England)	Mixed methods	241	NR	NR	NR	Inpatient	Patients & Staff	NR			IDD
Morris et al.	2021	UK	Quantitative	54	31	23	Mixed	Inpatient	Patients	32.5 [18–54]			ID with comorbid MH
Murphy et al.	2023	UK	Quantitative	98	98	0	Male	Mixed	Patient	NR [18–65]			ID or ASD
Novaco & Taylor	2014	UK (England)	Mixed methods	50	44	6	Mixed	Inpatient	Patients	30 [NR]			ID
Quinn et al.	2022	UK	Qualitative	4	NR	NR	NR	Inpatient	Patients & Staff	NR			ID
Sakdalan & Collier	2012	New Zealand	Mixed methods	3	3	0	Male	Inpatient	Patients	NR			ID with neurodiversity
Singh et al.	2011	USA	Mixed methods	3	3	0	Male	Inpatient	Patients	NR [23–34]			ID [& sex offenders]
Taylor & Novaco	2023	UK (England)	Quantitative	88	71	17	Mixed	Inpatient	Patients	32.6 [19–63]			ID
Taylor et al.	2017	UK	Mixed methods	80	NR	NR	NR	Inpatient	Patients & Staff	NR			ID with comorbid mental health
Taylor et al.	2012	UK	Qualitative	13	NR	NR	NR	Inpatient	Patients	NR			ID with comorbid mental health
Taylor et al.	2002b	UK (England)	Quantitative	14	8	6	Mixed	Inpatient	Patients	33.7 [20–48]			ID
Taylor et al.	2002a	UK	Quantitative	20	20	0	Male	Inpatient	Patients	NR [18–60]			ID
Taylor et al.	2005	UK (England)	Quantitative	36	36	0	Male	Inpatient	Patients	Intervention		Control	ID with comorbid mental health
										29.4 [18–60]		29.9 [18–60]	
Taylor et al.	2006	UK	Quantitative	6	0	6	Female	Inpatient	Patients	34.4 [20–48]			LD [& fire setting behaviour]
Taylor et al.	2003	UK (England)	Quantitative	66	NR	NR	NR	Mixed	Staff	NR			NR
Taylor et al.	2015	UK	Quantitative	50	44	6	Mixed	Inpatient	Patients	30 [NR]			ID with comorbid mental health
Tearle et al.	2020	UK	Qualitative	1	1	0	Male	Inpatient	Patients	29			ID
Thomas et al.	2005	UK (England)	Mixed methods	NR	NR	NR	NR	Inpatient	Patients	NR			ID
Travis & Sturmey	2013	USA	Quantitative	6	5	1	Mixed	Inpatient	Patients & Staff	Patients		Staff	ID with comorbid mental health
										NR [32–46]		NR [27–51]	
Wark & Gredecki	2023	UK	Qualitative	12	NR	NR	NR	Community	Staff	NR			NR
Whittle et al.	2021	UK (England)	Mixed methods	14	14	0	Male	Inpatients	Patients	18+			ID
Wooster et al.	2018	UK	Qualitative	40	40	0	Male	Inpatients	Patients	31.7 [18–65]			ID

*Note:* SO = sex offenders, OO = other types of offenders, F = female offenders, NR = not reported.

Studies were conducted primarily in the United Kingdom (*n* = 51, 91.07%) with a small number of studies conducted in the United States (*n* = 2, 3.57%), the Netherlands (*n* = 1, 1.78%) and New Zealand (n = 2, 3.57%). Twelve studies were qualitative, 31 were quantitative, and 13 were mixed methods. Reported mean sample size was 76.09, ranging from one (Ashworth et al. 2020) to 1281 (Chaplin et al. 2021). The study population included mostly patients (*n* = 45, 80.35%); seven included staff (12.5%); four included a combination of both patients and staff (7.14%). Only 13 studies reported the subgroup classifications of their sample sizes, which were omitted from Table 3 to maintain clarity. Nineteen studies (33.92%) were male‐only studies, compared with two (3.57%) with a female‐only population. Fourteen studies (25.0%) did not report the gender of the study population. The mean age of the population was 32.8 years, with age ranges reported between 18 and 79 years old. Regarding diagnosis, 32 studies reported a main diagnosis of ID of varying degrees; 18 studies reported a secondary diagnosis of a mental health condition; one study reported a secondary diagnosis of other forms of neurodiversity; and four with both. Thirteen studies gave no other details regarding the severity of ID or secondary diagnosis. For additional details, see Table 3.

The predominance of UK‐based studies (91%) likely reflects both the well‐established nature of specialist forensic ID services in the United Kingdom and the focus of national policy on service evaluation and discharge pathways in recent years. Our searches were international and without geographical limits; however, restricting to English‐language publications could have contributed to the high proportion of UK studies retrieved.

### 4.2 Q1: What Clinical Models of Care, Interventions and/or Service Models Are Utilised for People With ID in Forensic Mental Health Services?

We identified in total 49 models of care, service models and interventions (referred to as: ‘models’). Each evaluated programme, adaptation, intervention and so forth is considered a distinct approach within the scope of this review.

The identified models are first categorised under three concept categories: (i) patient/family; (ii) health practitioner/ward; and (iii) organisation/system level. A summary of key models of care and service models and interventions for people with ID in forensic mental health services is outlined in Table 4, but more detailed information is provided in Tables S1 and S2.

**TABLE 4:** Summary of key models of care and service models and interventions for people with ID in forensic mental health services.

Name of approach	Type	Key characteristics	Reported outcomes^a^
Discharge Pathway Protocol (Taylor et al. 2017)	Model of Care	Multi‐component, systemic approach to discharge preparation, role clarity, stakeholder coordination, post‐discharge follow‐up.	Reduced length of stay (LoS), reduced readmissions.
Care Pathway‐Based Approach (Devapriam et al. 2014)	Model of Care	Tier 4 core care pathway covering referral, assessment, treatment, discharge; coordinated by pathway nurse.	Reduced length of stay (LoS), increased admissions capacity, improved patient outcomes.
Psychological Treatment Pathway (Hickman et al. 2018)	Model of Care	Three‐stage, formulation‐led pathway and discharge plan, including BTSOP, BORTP, CBT, DBT, PBS.	Case studies show reduced risk and successful community discharge.
Community Forensic Learning Disability Team (Browning et al. 2016)	Service Model	Multidisciplinary outreach assessment, formulation, intervention and care coordination.	Reduced reconvictions, secure/out‐of‐area placements, severity of offending.
Fife Forensic Learning Disability Service (De Villiers and Doyle 2015)	Service Model	Community‐based, multi‐agency approach to assessment, intervention and risk management.	Increased discharges, reduced secure placements.
Specialist Court Liaison and Diversion Service (Chaplin et al. 2021)	Service Model	Court‐based ID screening, referral and diversion to health services.	Prevented inappropriate sentencing, improved service coordination.
Positive Behaviour Support (PBS) Plans (Whittle et al. 2021)	Intervention	Proactive behavioural support with staff training and well‐being focus.	Reduced restrictive practices, improved staff confidence/safety.
Brooklands Thinking Skills Offender Programme (BTSOP) (Hickman et al. 2017)	Intervention	Cognitive skills training for offenders with ID.	Reduced risk levels, improved discharge readiness.
CBT anger treatment (Novaco and Taylor 2015) (Taylor and Novaco 2023)	Intervention	Individualised CBT for anger management adapted for ID.	Reduced aggression, improved staff safety.
EQUIP (Langdon et al. 2013)	Intervention	Group‐based moral reasoning and social skills training.	Reduced incidents of aggression.
Interpersonal Art Psychotherapy (Hackett et al. 2020)	Intervention	Creative therapy addressing interpersonal causes of aggression.	Reduced aggression in patients with ID + schizophrenia.
Adapted SOTSEC‐ID (Sakdalan and Collier 2012)	Intervention	Sex offender treatment programme tailored for ID.	Reduced harmful/inappropriate sexual behaviours.
Talking Mats (Quinn et al. 2022)	Intervention	Visual communication tool to support patient choice and involvement.	Improved patient engagement and communication.

^a^ More details about all service models and interventions are provided in Tables S1 and S2.

Specific models linked to the patient/family concept category included the following:

- cognitive behavioural (individual or group based) therapy (CBT), for example, Fire‐Setter's treatment programme, (Taylor et al. 2006), CBT anger treatment, (Novaco and Taylor 2015), interpersonal art psychotherapy (Hackett et al. 2020) and ID‐adapted dialectical behaviour therapy (DBT) groups (Ashworth et al. 2021);
- wider programmes, for example, Community Forensic Learning Disability Team (CFT) (Browning et al. 2016) or service (Lindsay et al. 2006, 2013), Home Visit Programme within a forensic ID service (Cheshire et al. 2015), the Equipping Youth to Help One Another (EQUIP) Programme (Langdon et al. 2013; Tearle and Holt 2020) and the Brooklands Thinking Skills Offender Programme (BSOTP) (Hickman and Morris 2022; Hickman et al. 2017); and
- discrete/specific interventions, for example, ‘Mind Matters’, a group‐based psychoeducational programme (Ashworth et al. 2017), a neurofeedback intervention (Borghino et al. 2022) and a visual communication tool, for example, Talking Mats (Quinn et al. 2022).

Specific models linked to the health practitioner/ward concept category included the following:

- the use of assessment tools to evaluate patients' behaviour, symptoms or other factors, for example, the Emotional Problem Scale (EPS) (Ashworth and Mooney 2016), the Assessment of Interpersonal Risk (AIR) (Campbell and Mccue 2013), Essen Climate Evaluation Schema (EssenCES) (Chester et al. 2015), Brain Injury Screening Index (BISI) (Chester et al. 2018), the Dynamic Risk Outcome Scales (DROS) (Delforterie et al. 2023) and the Short‐Term Assessment of Risk and Treatability (START) (Inett et al. 2014);
- different approaches to planning the care together with the patients, for example, coproduction approaches to Dynamic Risk Assessment (Short Dynamic Risk Scale) (Morris et al. 2021), Management of Aggression Care Plan (MOACAP) (Thomas et al. 2005) and Positive Behaviour Support (PBS) plans (Whittle et al. 2021); and
- interventions targeting staff competence, for example, introductory workshop designed for direct care staff working with sex offenders with LD (Taylor et al. 2003) and reflective practice groups (Green and Cappleman 2023).

We acknowledge that a wide range of validated assessment tools was included in this concept category, as integral components of care delivery. Their use structures risk assessment, informs care planning, guides intervention choice or monitors treatment progress, thereby directly influencing the delivery and outcomes of a model of care.

Specific models linked to the organisation/system concept category focused on developing and/or evaluating services for people with ID, for example, a Specialist Court Liaison and Diversion service (Chaplin et al. 2021), Specialised Community Forensic Services (CFS) (Wark and Gredecki 2023), a Discharge Pathway Protocol (Taylor et al. 2017) and a specialist NHS Forensic service of a medium and low secure ward for men with ID (Wooster et al. 2018).

Furthermore, we categorised the identified models under three distinct but overlapping types of approaches to care for people with ID in forensic mental health services: models of care (MOC), service models and interventions:

- *4 Models of Care (MOC)*, which are structured, multi‐component frameworks that span the patient journey (e.g., Discharge Pathway Protocol, Care Pathway‐Based Approach, Psychological Treatment Pathway, Forensic Intellectual Disability Secure Services/[FIDSS] Model).
- *10 Service Models*, which are organisational approaches to delivering care, often at a community or system level (e.g., Community Forensic Learning Disability Teams, Fife Forensic Learning Disability Service, Specialist Court Liaison and Diversion Service); and
- *35 Interventions*, which are targeted therapeutic or practical approaches, often embedded within broader service models/models of care (e.g., BTSOP, adapted Dialectical Behaviour Therapy, Talking Mats communication tool).

Some approaches spanned multiple categories; for example, the Care Pathway‐Based Approach is a MOC that integrates specific interventions (e.g., Positive Behaviour Support) and operates within a specialist inpatient service model. Similarly, community forensic teams blended MOC principles with outreach service delivery.

Despite varied terminology, most approaches shared three core features: structured pathways, multidisciplinary working and person‐centred planning. The key differences lay in scope (whole service vs. targeted intervention), setting (inpatient vs. community) and degree of formalisation. Many interventions were inseparable from the MOCs or service models in which they were delivered, underscoring the blurred boundaries in the literature.

### 4.3 Q2: What Is Known About These Models' Potential Impact on Patient Outcomes, for example, Admissions, Length of Stay, Discharge, Improvement in Symptoms?

Thirty‐six studies reported patient‐level outcomes (effectiveness) related to admissions, length of stay (LoS), symptom change, discharge and community reintegration.

Models of Care: The Discharge Pathway Protocol (Taylor et al. 2017), Care Pathway‐Based Approach (Devapriam et al. 2014) and Psychological Treatment Pathway (Hickman et al. 2018) demonstrated reductions in LoS, timely discharges and improved post‐discharge stability. These models emphasised clarity of roles, coordinated/structured planning and active post‐discharge support.

Service Models: Community Forensic Learning Disability Teams (Browning et al. 2016) and the Fife Forensic Learning Disability Service (De Villiers and Doyle 2015) reported reductions in secure placements, reconvictions and severity of offending behaviour, highlighting the value of sustained community‐based multidisciplinary engagement.

Interventions: Programmes such as BTSOP (Hickman et al. 2017), CBT anger treatment (Novaco and Taylor 2015) and adapted fire‐setter or sexual offender programmes (Taylor et al. 2006; Borghino et al. 2022) showed improvements in treatment engagement, reduced aggression and lower assessed risk.

Evidence for effectiveness was strongest for pathway‐based MOCs with integrated discharge planning, though high‐quality comparative studies were rare. Service models appeared most effective in sustaining community living, while targeted interventions often addressed specific risks, but were dependent on broader service and pathway structures for impact.

### 4.4 Q3: What Is Known About These Models' Potential Impact on Patient Safety, for Example, Self‐Harm, Violence and the use of Restrictive Practices?

Twenty‐six studies examined outcomes such as the use of restrictive practices, incidents of aggression and self‐harm.

Models of Care: The Psychological Treatment pathway (Hickman et al. 2018) showed a reduction in aggressive and offending behaviour.

Service Models: Specialist community forensic teams and trauma‐informed approaches (Browning et al. 2016; Wark and Gredecki 2023) reported reduced risk behaviours, reoffending and reduced reliance on the use of restrictive practices.

Interventions: Interpersonal Art Psychotherapy (Hackett et al. 2020), EQUIP (Langdon et al. 2013) and individual CBT anger treatment (Novaco and Taylor 2015) reduced aggression and harmful behaviours. Outcomes varied by population and setting; for example, ID‐adapted DBT was less effective for aggression reduction in some inpatient male populations (Craven and Shelton 2020).

Safety improvements were most robust when interventions were embedded within a whole‐system approach emphasising positive engagement, staff training and trauma‐informed principles. Standalone interventions had variable effects unless supported by broader organisational change.

### 4.5 Q4: What Is Known About These Models' Potential Impact on Staff Outcomes, Attributes and Needs of Staff Delivering Care to People With ID in Forensic Mental Health Services?

Seventeen studies explored staff‐focused outcomes, including safety, retention, confidence and training needs.

Models of Care: Implementing structured care improved staff retention, perceived safety and team cohesion (Devapriam et al. 2014; Taylor et al. 2017).

Service Models: Court liaison services and multidisciplinary community teams enhanced interprofessional collaboration and reduced perceived role conflict (Chaplin et al. 2021).

Interventions: Training in risk assessment tools (e.g., AIR; Campbell and McCue 2013) and offender‐specific programmes improved staff knowledge, confidence and decision‐making. Reflective Practice Groups (Green and Cappleman 2023) and psychosocial interventions (Isherwood et al. 2006) also improved team relationships and coping with stress, though the need for further training and adaptation to ID populations was frequently noted.

Staff outcomes improved when MOCs embedded structured training, reflective spaces and collaborative practices. However, sustainability depended on organisational support, workload management and appropriate skill‐mix.

### 4.6 Q5: What Is Known About Patients and Family Members' Expectations and Experiences of Care in Forensic Mental Health Services?

Thirteen studies addressed patient and family perspectives.

Models of Care: Pathways that incorporated patient involvement in planning (e.g., coproduced care plans) were associated with greater perceived ownership, improved coping and enhanced trust.

Service Models: Community‐based services facilitated family contact more easily than secure inpatient settings, supporting continuity of relationships and perceived quality of life.

Interventions: Communication tools (e.g., Talking Mats) (Quinn et al. 2022) and structured engagement programmes improved patients' ability to express preferences and feel heard, though some patients still perceived care as intrusive or insufficiently relational (De Villiers and Doyle 2015).

Positive patient and family experiences were linked to consistent communication, involvement in decision‐making and maintenance of social connections. Barriers were most evident in high‐security settings with limited visiting and peer contact opportunities.

## 5 Discussion

In this scoping review, we identified and categorised evidence across three concept categories (patient/family; health practitioner/ward; organisation/system) and three distinct but related approaches to care for people with ID in forensic mental health services (clinical models of care, broader service models and specific interventions). This distinction addresses a recurrent gap in the literature, where these terms are often used interchangeably despite their different implications for practice and policy. By applying consistent definitions and classification criteria, we were able to map both the diversity of approaches used in forensic mental health services for adults with intellectual disabilities (ID) and the outcomes they have reported.

We adapted the framework developed by (Morrissey et al. 2017) to report four key outcome domains related to the identified models: (i) effectiveness, (ii) patient safety, (iii) patient and family/carer experience and (iv) staff outcomes, skills and attributes.

### 5.1 Effectiveness

There are four models of care (‘best practice’ models) that stand out within inpatient settings in this area:

1. the Discharge Pathway Protocol, a multifaceted and systemic approach to discharge preparation and planning, that was implemented and tested by (Taylor et al. 2017) in a locked rehabilitation unit part of a specialist secure service for men with ID in North East England, UK. The protocol includes core components delivered pre‐ and post‐discharge. These components are flexible and can be implemented at a pace or order to suit the individual patient's needs. Implementing the protocol was found to be beneficial in reducing length of stay, facilitating timely discharges and reducing readmission rates. In particular, four key specifics of the protocol were found to be useful: clarity of the process (steps needing completion) and roles (what needs to be done, when and by whom); partnership working (bringing together of stakeholders working alongside and toward common goals); specialist risk management training (improving staff's skills and confidence regarding perceived risks for a smooth transition for individuals moving from a hospital to a community placement); and post‐discharge follow‐up (essential outreach support to provide continuity post‐discharge to help individuals to reintegrate successfully in the community);
2. the Care Pathway‐Based Approach, a Tier 4 core care pathway for referral, assessment, treatment and discharge within a specialist inpatient unit for adults with intellectual disabilities in Leicester, England, UK. The approach had a ‘lean’ straightforward pathway including a referral checklist, admission procedures, formulation and multidisciplinary meetings, timely assessments and interventions, discharge planning and outcome measurements. A pathway coordinator (band six nurse) had the key role of ensuring progress of patient journey through the pathway through proactive and joint working with the relevant agencies and professionals. This was found to significantly reduce the average length of stay, as well as improving the turnover of more patients, increased capacity for admissions to the unit and better outcomes for patients (e.g., HONOS‐LD scores) (Devapriam et al. 2014).
3. the Psychological Treatment Pathway (Hickman et al. 2018), implemented in a male medium and low secure ID service in Birmingham, England, UK. The pathway comprises three key stages: Stage 1 is concerned with treatment direction setting, risk minimisation and engagement, initial assessments; Stage 2 is the active treatment phase; and Stage 3 is concerned with final discharge processes/exit preparation. Key interventions in the active treatment stage include: the BTSOP (Hickman et al. 2017), the Brooklands Relationships Offender Treatment Programme (BORTP) (Murphy 2014), CBT combined with the Good Lives Model (Ward 2002), DBT, CBT for substance abuse relapse prevention, PBS. Based on case studies, this pathway demonstrated subsequent reductions in assessed risk and successful community discharge. While it is difficult to generalise given the small numbers, the case study approach is showing that a formulation led pathway can be both clear and structured while being flexible and individualised to each person's needs.
4. the FIDSS Model of Care (Duff et al. 2023) was implemented in a specialist forensic ID secure care unit in Aotearoa (New Zealand). Though not yet tested for effectiveness, FIDSS offers a holistic and culturally sensitive approach that could be adapted for diverse populations. It identifies the core components and principles of a good model of care that is (i) centred around the person and their wider social network and (ii) focused on safety, rehabilitation and quality of life. The model uses a responsive approach to contemporary needs based on a fundamental requirement to understand why the individual has been admitted into hospital and what needs to be done, so they can successfully move forward. The Mason FIDSS Approach combines PBS (Positive Behaviour Support) (Hickman et al. 2018), the Good Lives model (Aust 2010), Trauma Informed Care (Chester et al. 2017) and a holistic approach to health, taking into account (in this case) the cultural and intergenerational needs of the Māori population. While adapted with and for Māori individuals with ID in forensic services, this model is generalisable to other cultures. The model weaves together key elements to optimise specialist forensic care for people with ID which could be considered as building blocks of any models developed for this population:

- care values—the need for expression of human rights, receiving compassion, having choices, being able to connect with social networks, succeeding in goals and being treated with respect and honesty;
- quality of life, agency and autonomy—supported through an appropriate, homely, personalised physical environment (as people tend to stay in these hospitals for a long time, and design has been shown to improve clinical outcomes and safety and reducing stress for both patients and staff users and staff (Ulrich 2006)) and an appropriate social environment that helps maintaining agency and autonomy, by protecting residual liberties for individuals with ID in forensic services, especially in secure settings, where safety trumps freedom; and
- appropriate skills, knowledge and attributes of staff, part of a diverse multidisciplinary team—the delivery and effectiveness of any model of care depends greatly on the staff team. Without the right training and support for staff, quality of care will be negatively impacted, while the fidelity and delivery of the model could be compromised.

Table 5 summarises the four best practice models, highlighting their defining features and the outcomes reported across the included studies.

**TABLE 5:** Summary characteristics of four ‘best practice’ models of care in forensic mental health services for people with intellectual disabilities.

Model	Setting & scope	Core components	Reported outcomes
Discharge Pathway Protocol (Taylor et al. 2017)	Locked rehabilitation unit within specialist secure service, UK	Flexible, multi‐stage discharge process; clarity of steps and roles; partnership working; specialist risk management training; post‐discharge follow‐up.	Reduced length of stay (LoS), perceived (by staff) safe and effective discharge, successful transitions with continued care, reduced readmissions.
Care Pathway‐Based Approach (Devapriam et al. 2014)	Specialist inpatient unit, UK	Tier 4 pathway with referral checklist, admission process, formulation meetings, timely assessments/interventions, discharge planning; pathway nurse coordinator.	Reduced length of stay (LoS), increased admissions capacity, improved patient outcomes (HONOS‐LD), discharged more quickly and safely.
Psychological Treatment Pathway (Hickman et al. 2018)	Medium/low secure ID service, UK	Three stages: direction setting and risk minimisation, active treatment, discharge preparation; integrates BTSOP, BORTP, CBT, DBT, PBS.	Reduced assessed risk (HCR‐20, SCR‐20, HONOS), reduction in aggressive and offending behaviour, increased successful community discharge (case studies).
Forensic Intellectual Disability Secure Services (FIDSS) Model (Duff et al. 2023)	Specialist forensic ID secure care unit, New Zealand	Holistic, culturally sensitive (Māori‐focused); integrates PBS, Good Lives, trauma‐informed care; emphasises person‐centred care, rehabilitation, quality of life.	Not yet formally evaluated; identified as promising framework adaptable to other populations.

Effective discharge needs to incorporate continuation of support in the community (where appropriate). There is also evidence to support benefits and outcomes linked to interventions delivered by specialist community‐based services—for example, the Community Forensic Learning Disability Team (CFT) (Browning et al. 2016) and Fife Forensic Learning Disability Service (FFLDS) (De Villiers and Doyle 2015). Using a comprehensive multidisciplinary multiagency approach to engagement, assessment, formulation and intervention, these provide the evidence for a reduction in people residing in secure settings, reductions in convictions and severity of offending behaviours.

### 5.2 Patient Safety

The studies reviewed highlight various interventions showing reductions in aggression, restrictive practices and improving safety for individuals with ID in forensic services, for example, the ‘Flip the Triangle’ strategy (Whittle et al. 2021); Interpersonal Art Psychotherapy (Hackett et al. 2020) and EQUIP (Langdon et al. 2013). Individual‐based CBT anger treatment and adapted Sex Offender Treatment Services Collaborative—Intellectual Disabilities (SOTSEC‐ID) demonstrated notable declines in harmful and inappropriate behaviours (Novaco and Taylor 2015; Taylor et al. 2016, 2005).

### 5.3 Staff Outcomes, Skills and Attributes

Various interventions have shown promise in reducing staff harm, improving recruitment and retention, enhancing care quality and addressing staff training and emotional needs. Individual CBT and anger‐focused CBT programmes significantly reduced assaults/staff harm (Novaco and Taylor 2015; Taylor et al. 2002). The PRISM protocol and service appraisals positively impacted staff retention (De Villiers and Johnstone 2024; Chaplin et al. 2011). Training workshops and tools like the ‘Assessment of personal risk’ (Campbell and Mccue 2013) improved staff knowledge, attitudes and decision‐making. Interventions like Reflective Practice Groups (Green and Cappleman 2023) and Psychosocial Interventions (Isherwood et al. 2006) improved relationships with patients and mutual trust, as well as boosting staff confidence and ability to cope with stress—also indicated by (McKinnon et al. 2024) who explored the implementation of a Work‐to‐Wellbeing intervention within secure Intellectual and Developmental Disabilities (IDD) services. Many studies, however, highlight the need for more and/or appropriate training for staff working with people with ID in forensic services (e.g., Chester et al. 2015; Ashworth and Mooney 2016; Taylor et al. 2012; Chaplin et al. 2024; Morris et al. 2021; Green and Cappleman 2023; McKinnon et al. 2024).

For a summary of outcomes of models of care, service models and interventions, see Table 6 (detailed information included in Tables S1 and S2).

**TABLE 6:** Outcomes of models of care, service models and interventions by domain.

Domain	Common components	Reported benefits	Limitations/gaps
Effectiveness	Structured pathways; clear roles/responsibilities; integrated discharge planning; community follow‐up.	Reduced length of stay, increased timely discharges, reduced readmissions, improved functioning (HONOS‐LD).	Few RCTs or long‐term follow‐ups; limited generalisability beyond UK.
Patient safety	Trauma‐informed care; proactive behavioural support; staff training in risk management; therapeutic engagement.	Reduced restrictive practices, aggression, harmful behaviours; improved ward climate.	Mixed findings for some populations (e.g., DBT for aggression in male inpatients).
Patient/family experience	Coproduction of care plans; communication tools; facilitation of family contact.	Increased patient ownership, trust, dignity; improved quality of life.	Challenges in secure settings (limited visiting, peer relationships); some care perceived as intrusive.
Staff outcomes	Staff training; reflective practice; multidisciplinary collaboration; supportive leadership.	Improved safety, retention, confidence, team cohesion, decision‐making.	Need for ongoing training; sustainability linked to organisational support and resources.

*Note:* More details are provided in Tables S1 and S2.

### 5.4 Patient and Family/Carer Experience

Fewer studies have captured these experiences. The studies emphasise the importance of patient involvement in their own care (Ashworth et al. 2020; Quinn et al. 2022; Thomas et al. 2005), highlighting benefits such as improved coping strategies, risk management and a sense of ownership and responsibility. Regular communication with patients (Chaplin et al. 2011) fosters dignity, respect and prevents negative impacts from others' behaviour. Maintaining family contact in secure settings is challenging but crucial for improving patients' quality of life (Cheshire et al. 2015).

### 5.5 Limitations and Implications for Research, Policy and Practice

This extensive scoping review was best placed to map out a wide range of available evidence regarding models of care for people with ID in forensic services; however, it should be noted that this has its limitations, as the studies included were not formally assessed for quality, which might reduce the strength of the evidence when it comes to informing policy or practice change.

Models of care are usually criticised for their focus on principles rather than clear goals, pathways, processes, treatments, evaluation and logic models (Kennedy 2022). Evidence suggests that clinical diagnosis or categories of offending behaviour are not significantly associated with length of stay (Grann et al. 2008). Individuals in these settings might appear homogeneous in this regard (diagnosis and/or offence category), but they respond differently to treatment and their movement through the care pathway, including length of stay. Given the current focus on improving efficiency and productivity within healthcare services in the United Kingdom and elsewhere, it is important to consider that the funding/commissioning of services for people with LD in forensic services is not solely based on diagnosis/offence category.

Larger studies are needed in the forensic ID field to explore the complexity of these settings and the predictors of successful discharge and reduced length of stay. Long‐term outcome research for this population is lacking, despite some of the included studies suggesting that the clinical needs of this patient population group remain following discharge. Studies need to focus on larger samples and longer follow‐up periods to capture appropriate outcomes and the mechanisms by which these are achieved (e.g., using realist evaluations and/or implementation science).

The paucity of validated tools for measuring outcomes for people with ID in forensic settings makes it particularly difficult to test the effectiveness of interventions for this population. The limited use of validated outcome measures constrains the comparability and robustness of findings, making it challenging for services to benchmark performance or reliably assess the effectiveness of models of care. Future research should prioritise the development, adaptation and consistent use of acceptable and psychometrically sound tools suitable for forensic ID populations.

Gender inequality appears to be another significant limitation, both in terms of service provision and research capturing the evidence base. The case load for models or interventions reported in this review is overwhelmingly male, much higher than the percentage of females in the forensic ID inpatient population. The under‐reporting of gender differences limits our understanding of whether models of care are equally effective for men and women or require tailoring to meet gender‐specific needs; future evaluations should ensure adequate representation and analysis across genders.

There is a lack of studies including patients and/or family members. There is an urgent need to flip the narrative and bring the voice/input of the people with lived experience to the forefront of delivering services, co‐developing care, outcomes and research that captures their experiences and their progress through forensic services.

In addition, the predominance of UK‐based studies raises questions about the transferability of findings to other forensic healthcare settings, underlining the need for cross‐national studies to test the adaptability of best practice models in different policy and service contexts. Addressing these gaps will not only strengthen the evidence base, but also support more equitable, effective and contextually relevant service development. A further limitation relates to the historical conflation of models of care, service models and interventions within primary studies, which may have obscured differences in scope, required resources and anticipated outcomes. Our categorisation approach aimed to address this, but future research should adopt and report consistent terminology to enhance comparability and translation into service development.

## 6 Conclusion

The strongest evidence from this review relates to four ‘best practice’ models of care: the Discharge Pathway Protocol, the Care Pathway‐Based Approach, the Psychological Treatment Pathway and the FIDSS Model. These approaches share a focus on structured pathways, multidisciplinary teamwork and person‐centred care, and have been linked to improved discharge outcomes, enhanced patient experience, and, in the case of the FIDSS model, culturally sensitive practice. Successful implementation requires appropriate staff training and support, integration with community‐based services to ensure continuity of care, and active involvement of patients and families in goal‐setting and care planning. For policymakers, these findings emphasise the importance of investing in evidence‐informed pathways and robust outcome monitoring; for clinicians, they highlight the value of embedding structured risk assessment, goal‐focused interventions and collaborative, person‐centred planning into routine practice. Together, these models offer practical frameworks for developing and refining services to meet the needs of this population.

## Conflicts of Interest

The authors declare no conflicts of interest.

## Supporting information


**Table S1:** Outcomes of models of care/service models/interventions for people with ID involved with forensic mental health services.
**Table S2:** Details of studies reporting on all four outcomes (further breakdown from Supplementary Table S1).

## Data Availability

Data sharing is not applicable to this article as no datasets were generated or analysed during the current study.
