# Supplementary material for: Clinical Models of Care for Adults With Intellectual Disabilities in Forensic Mental Health Services: A Scoping Review
Source: J Intellect Disabil Res. 2025 Oct 13;70(3):242–61. doi: 10.1111/jir.70048 (PMC12872379; doi:10.1111/jir.70048)
Supplement: Supplementary file 1 — Table S1: Outcomes of models of care/service models/interventions for people with ID involved with forensic mental health services. Table S2: Details of studies reporting on all four outcomes (further breakdown from Supplementary Table S1). [file JIR-70-242-s001.docx]

**Supplementary Table S1. Outcomes of models of care/service models/interventions for people with ID involved with forensic mental health services**

|  | Type of approach | Concept category | | | Outcome category | | | |
| --- | --- | --- | --- | --- | --- | --- | --- | --- |
| Model of care/service model/intervention |  | Patient/  family | Practitioner/  ward | Organisation/  system | Effectiveness | Patient Safety | Patient & family/carer  experiences | Staff outcomes, skills & attributes |
| A 10-point treatment program  Alexander et al., 2011 | Intervention | x |  |  | ↑ | ↑ | = | = |
| Individual intervention based upon a Cognitive Behaviour Therapy (CBT) model for depression  Ashworth et al., 2020 | Intervention | x |  |  | ↑ ↓ | = | ↑ | = |
| "Mind Matters" - a group-based psychoeducational programme  Ashworth et al., 2017 | Intervention | x |  |  | ↑ | = | ↑ | ↑ |
| The emotional problem scales (EPS; Prout and Strohmer, 1991) - used within a structured therapeutic programme to assess emotional difficulties in people with ID, with results feeding directly into tailored intervention planning and review  Ashworth & Mooney, 2016 | Intervention |  | x |  | = | = | = | = |
| Training programme for staff around the ‘I Can Feel Good’ programme.  Ashworth et al., 2016 | Intervention |  | x | x | = | = | = | ↑ |
| DBT groups   - ‘I Can Feel Good’ programme, adapted dialectical behaviour therapy (DBT) informed group-based skills programme. - Intellectual disability-adapted dialectical behaviour therapy (DBT) skills group sessions.   Ashworth et al., 2021  Browne et al., 2019  Craven & Shelton, 2020 | Intervention | x  x  x |  |  | ↑  =  ↑ | ↑  ↑  ↑ | =  ↑  = | =  =  = |
| Neurofeedback intervention  Borghino et al., 2022 | Intervention | x |  |  | ↑ | ↑ | ↑ | = |
| Community Forensic Learning Disability Team (CFT) delivered interventions (psychology/speech and language/occupational therapy & offence specific interventions)  Browning et al., 2016 | Service Model | x | x | x | ↑ | ↑ | = | ↑ |
| The Assessment of Interpersonal Risk (AIR) - implemented as part of a multidisciplinary risk management framework to guide care planning, security level decisions, and therapeutic focus  Campbell & McCue., 2013 | Intervention |  | x |  | = | ↑ | = | = |
| Specialist inpatient service and part of the Mental Health and Intellectual disabilities (MH-LD) care pathway  Chaplin et al., 2011 | Service Model | x | x | x | Nil outcome measures (recommendations for future) | / | / | / |
| Specialist Court liaison and diversion service  Chaplin et al., 2021 | Service Model |  |  | x | ↑ | = | = | = |
| Specialist ND L&D service Chaplin et al., 2024 | Service Model |  | x | x | ↑ | = | ↑ | ↑ |
| Home visit programme within a forensic intellectual disability service  Cheshire et al., 2015 | Intervention | x |  | x | = | = | ↑ | = |
| Essen Climate Evaluation Schema (EssenCES) - used to measure and improve ward climate as part of service-wide quality improvement and safety initiatives  Chester et al., 2015 | Intervention | x | x |  | = | = | = | = |
| Brain Injury Screening Index (BISI©) - embedded into admission assessment protocols to identify brain injury needs early, influencing care pathway design and intervention selection  Chester et al., 2018 | Intervention |  | x |  | = | = | = | ↑ |
| The Dynamic Risk Outcome Scales (DROS) - used within dynamic risk assessment models to monitor treatment impact and readiness for step-down or discharge  Delforterie et al., 2023 | Intervention | x | x |  | =  (however, could be used to predict recidivism) | = | = | = |
| A core care pathway for referral, assessment, treatment and discharge  Devapriam et al., 2014 | Model of Care |  |  | x | ↑  Although increased appropriate admissions | = | = | ↑ |
| Fife Forensic Learning Disability Service (FFLDS)  de Villiers & Doyle, 2015 | Service Model | x | x | x | ↑ | = | = | = |
| PRISM Protocol to reduce violent incidents de Villiers & Johnstone, 2024 | Intervention | x | x |  | ↑ | ↑ | = | ↑ |
| Model of care for a specialist forensic intellectual disability secure care unit in Aotearoa (New Zealand)  Duff et al., 2023 | Model of Care | x | x | x | Nil outcome measures | / | / | / |
| Reflective Practice Groups (RPGs) in an Inpatient Intellectual Disability Service  Green & Cappleman, 2023 | Intervention |  | x |  | = | = | = | ↑ |
| Interpersonal art psychotherapy  Hackett et al., 2020 | Intervention | x |  |  | ↑ | ↑ | ↑ | = |
| Leavers Preparation Group  Hickman et al., 2018a | Intervention | x |  |  | ↑ | = | ↑ | = |
| Brooklands Thinking Skills Offender Programme (BSOTP)  Hickman & Morris, 2022  Hickman 2017 | Intervention | x  x |  |  | ↑  ↑ | =  = | =  = | =  = |
| Psychological treatment pathway  Hickman et al., 2018b | Model of Care | x | x | x | ↑ | ↑ | = | = |
| Short-Term Assessment of Risk and Treatability (START) - integrated into ongoing dynamic risk management to inform multidisciplinary decision-making about intervention intensity and discharge readiness  Inett et al., 2014 | Intervention |  | x |  | =  (however, could be used to predict physical aggression, verbal aggression, self-harm) | = | = | = |
| Psychosocial interventions (PSI framework) Isherwood et al., 2006 | Intervention | x | x |  | Nil outcome measures | / | / | / |
| EQUIP groups   - The Equipping Youth to Help One Another (EQUIP) Programme - Adapted Equipping Youth to help One Another (EQUIP) programme   Langdon et al., 2013  Tearle et al., 2020 | Intervention | x  x |  |  | ↑  ↑ | ↑  ↑ | =  ↑ | =  = |
| Violence risk assessment appraisal guide (VRAG), Static-99 and level of service need/security  Lindsay et al., 2010 | Intervention | x | x |  | = | = | = | = |
| A comprehensive community service for offenders with ID  Lindsay et al., 2006  Lindsay et al., 2013 | Service Model | x  x | x  x | x  x | ↑  ↑ | =  ↑ | =  = | =  = |
| W2W (Work-to-Wellbeing) within secure Intellectual and Developmental Disabilities (IDD) services.  McKinnon et al., 2024 | Intervention | x | x |  | = | = | = | ↓ |
| Coproduction approaches to dynamic risk assessment (Short Dynamic Risk Scale) - a collaborative risk assessment method involving patients and staff, forming part of proactive behaviour support and care planning  Morris et al., 2021 | Intervention | x | x |  | = | = | ↑ | ↑ |
| Group CBT for men who have harmful sexual behaviour (SOTSEC-ID)  Murphy et al., 2023 | Intervention | x |  |  | ↑ | = | = | = |
| Individual-based CBT anger treatment  Taylor & Novaco, 2023  Novaco & Taylor, 2015  Taylor et al., 2016  Taylor et al., 2005  Taylor et al., 2002a | Intervention | x  x  x  x  x |  |  | ↑  ↑  ↑  ↑  ↑ | ↑  ↑  ↑  ↑  ↑ | =  =  =  =  = | =  =  =  =  = |
| Visual communication tool Talking Mats  Quinn et al., 2022 | Intervention | x | x |  | = | = | ↑ | ↑ |
| SAFE-ID, an Adapted Sex Offender Treatment Services Collaborative – Intellectual Disabilities (SOTSEC-ID) programme, individual and group-based  Sakdalan & Collier, 2012 | Service Model | x |  |  | ↑ | ↑ | ↑ | ↑ |
| Meditation on the Soles of the Feet, and a Mindful Observation of Thoughts meditation- procedure to control deviant sexual arousal  Singh et al., 2011 | Intervention | x |  |  | ↑ | ↑ | ↑ | = |
| Introductory workshop designed for direct care staff working with sex offenders with ID  Taylor et al., 2003 | Intervention |  | x |  | = | = | = | ↑ |
| Discharge pathway protocol  Taylor et al., 2017 | Model of Care | x | x | x | ↑ | = | = | ↑ |
| Group-based CBT   - Fire-Setter’s treatment programme - A group intervention for convicted arsonists   Taylor et al., 2006  Taylor et al., 2002b | Intervention | x  x |  |  | ↑  ↑ | ↑  ↑ | =  = | =  = |
| An adapted therapeutic community model  Taylor et al., 2012 | Service Model | x | x |  | Nil outcome measures (No empirical data reported) | / | / | / |
| Management of aggression care plan (MOACAP)  Thomas et al., 2005 | Intervention | x | x |  | ↑ | ↑ | = | ↑ |
| Behavioural skills training (BST)  Travis & Sturmey, 2013 | Intervention | x | x |  | ↑ | ↑ | = | = |
| Specialised community forensic services (CFS)  Wark & Gredecki, 2023 | Service Model |  |  | x | = | ↑ | = | ↑ |
| Positive behaviour support (PBS) plans  Whittle et al., 2021 | Intervention | x | x | x | ↑ | ↑ | = | ↑ |
| NHS Forensic service of a medium and low secure ward for men with ID  Wooster et al., 2018 | Service Model |  |  | x | ↑ | = | = | = |

***Note: ‘***↑’ indicates positive outcome, ‘↓’ negative outcome, ‘=’ indicate no changes/no outcome, ‘/’ indicates Nil outcome measure, ‘x’ indicates the intervention categorisation

## **Supplementary Table S2. Details of studies reporting on all four outcomes(further breakdown from Supplementary Table S1)**

| **Outcomes** | **Studies** | **Model of Care/** **Service Model/Intervention** | **Findings** |
| --- | --- | --- | --- |
| **Effectiveness outcomes**  **(n=36 studies)** |  |  |  |
| a. Length of stay (n=4) | Alexander et al, 2011 | 10-point treatment programme | LoS reduced |
|  | Devapriam et al., 2014 | Care pathway-based approach | LoS reduced |
|  | Taylor et al., 2017 | Discharge pathway protocol | LoS reduced |
|  | Wooster et al., 2018 | NHS Forensic Service | LoS reduced |
| b. Admission or re-admission  (n=4) | Browning et al., 2016 | Community Forensic Team (CFT) | Prevented readmission in secure services |
|  | Devapriam et al., 2014 | Care pathway-based approach | Increased capacity for admissions |
|  | Wooster et al., 2018 | NHS forensic service | Readmission to low security levels |
|  | Taylor et al., 2017 | Discharge pathway protocol | Low re-admission rates |
| c. Discharge pathway/plan/placement and recommendations  (n=14) | Sakdalan & Collier, 2012 | SAFE-ID | Eventually transitioned or taken out of the legal order |
|  | Alexander et al., 2011 | 10-point treatment programme | Discharged to lower observational levels |
|  | Browning et al., 2016 | CFT | Discharge planning, appropriate placements |
|  | de Villiers & Doyle, 2015 | FFLDS | Majority discharged to community. |
|  | Delforterie et al., 2023 | DROS recidivism subscale | Predict safety to discharge along with recidivism |
|  | Devapriam et al., 2014 | Care pathway-based approach | Discharged more quickly and safely |
|  | Hickman et al., 2018a | Leavers’ Preparation Group | Beneficial in preparing patients for discharge/transfer |
|  | Hickman & Morris 2022 | BSOTP | Successful discharge/transfer to community |
|  | Hickman et al., 2017 | BSOTP | Successful discharge/transfer to community |
|  | Hickman et al., 2018b | Psychological Treatment Pathway | Structured discharge plan for MSU and LSU |
|  | Taylor et al., 2006 | Fire setters’ Treatment Programme | Transferred to community |
|  | Taylor et al., 2017 | Discharge pathway protocol | Successful transitions with continued care |
|  | Tearle et al., 2020 | EQUIP (1) | Transition and resettlement |
|  | Wooster et al., 2018 | NHS Forensic Service | Discharged to community and supported living environment |
| d. Re-offending/re-conviction/relapse  (n=9) | Borghino et al., 2022 | Neurofeedback intervention | Reduced risk to others, incidents of sexual violence |
|  | Ashworth et al., 2020 | CBT | Mentions relapse prevention for depressed inpatients |
|  | Browning et al., 2016 | CFT | Reduction in recidivism and reconviction |
|  | Delforterie et al., 2023 | DROS subscale | Predicts recidivism |
|  | Hickman & Morris, 2022 | BSOTP | No recidivism in follow up |
|  | Hickman et al., 2017 | BSOTP | Reduced risk of recidivism |
|  | Lindsay et al., 2013 | A comprehensive community service for offenders with ID | 90% reduction in offending rates although recidivism rates are disappointing in the first 12 months. |
|  | Lindsay et al., 2006 | A comprehensive community service for offenders with ID | Reports offences in different groups. The study indicates significant reductions in recidivism, especially for the Sex Offender (SO) and Female Offender (F) groups. |
|  | Taylor et al., 2006 | Group based Fire setters’ Treatment Programme | No re-offending in 2-year follow-up study |
| e. Progress (determined by response to treatment, reduction in risk and considered safe for discharge or transfer)  (n=28) | Borghino et al., 2022 | Neurofeedback interventions | Reduced offensive behaviour, reduced symptoms such as depression, more community leaves |
|  | Sakdalan & Collier, 2012 | SAFE-ID | Sexual knowledge, cognitive distortions, dynamic risk factors |
|  | Ashworth et al., 2020 | CBT for depression | Emotional and behavioural  Therapeutic engagement  (Increased anxiety and depression symptoms were observed towards the end of the intervention) |
|  | Ashworth et al., 2017 | Mind matters | High attendance and engagement, social skills |
|  | Ashworth et al., 2021 | ‘I can feel good’ programme | Skills acquisition, people skills |
|  | Craven & Shelton, 2020 | Intellectual disability-adapted dialectical behaviour therapy (DBT) skills group sessions | Emotional regulation and anger management not significant |
|  | Chaplin et al., 2021 | CMH+NDD | Better referrals and outcomes |
|  | Chaplin et al., 2024 | Specialist ND L&D service | Alternative to prison for ND population |
|  | De Villiers & Johnstone, 2024 | PRISM protocol for violence reduction | Reduction in risk |
|  | Delforterie et al., 2023 | DROS subscale | Progress towards predicting risk |
|  | Hackett et al., 2020 | Interpersonal art psychotherapy | Aggression and distress related symptoms |
|  | Hickman et al., 2018a | Leavers’ preparation group | Reduced anxiety and readiness to discharge |
|  | Hickman et al., 2017 | BSOTP | Improved internal and external locus of control, problem-solving skills |
|  | Hickman et al., 2018b | Psychological treatment pathway | Risk reduction |
|  | Langdon et al., 2013 | EQUIP | Moral reasoning, distorted cognition, problem-solving |
|  | Murphy et al., 2023 | SOTSEC-ID | Sexual knowledge, victim empathy, cognitive distortions maintained at 6 months follow-up |
|  | Novaco & Taylor, 2015 | Individual-based CBT anger treatment | Aggressive behaviour |
|  | Singh et al., 2011 | Meditation on the Soles of the Feet, and a Mindful Observation of Thoughts meditation | Self-control on sexual deviant arousals |
|  | Taylor & Novaco, 2023 | Individual-based CBT anger treatment | Aggressive behaviour |
|  | Taylor et al., 2006 | Fire setters’ treatment programme | Self-esteem, anger, and depression post-treatment |
|  | Taylor et al., 2002a | Individual-based CBT anger treatment | Aggressive behaviour |
|  | Taylor et al., 2002b | A group intervention for convicted arsonists | Self-rated anger |
|  | Taylor et al., 2005 | Individual-based CBT anger treatment | Aggressive behaviour |
|  | Taylor et al., 2016 | Individual-based CBT anger treatment | Aggressive and violent incidents |
|  | Tearle et al., 2020 | EQUIP | Self-esteem, anger, and depression post-treatment |
|  | Travis & Sturmey, 2013 | BST | Aggressive responses and replacement behaviours |
|  | Wark & Gredecki, 2023 | CFS | Reliance on restraint techniques |
|  | Whittle et al., 2021 | PBS | Positive and proactive interventions and staff confidence |
| f. Use of tools, e.g., HONOS, HONOS-LD, HONOS-secure, BPI, PASS-AD, GAF, ABC, CGI, TAG, SCL-90-R, Reiss Screen for Maladaptive Behaviour, Brief Psychiatric Rating Scale, DASH scale, VAS, PIRMA, GSI, SAB, HCR-20  (n=2) | Devapriam et al., 2014 | Care pathway-based approach | HONOS-LD scores improved |
|  | Hickman et al., 2018b | Psychological treatment pathway | Improvement in risk assessment through HCR-20, SCR-20, HONOS |
| **Patient safety outcomes**  **(n=26)** |  |  |  |
| a. Use of restrictive practices (e.g., restraint, seclusion, observation, PRN)  (n=2) | Wark & Gredecki, 2023 | CFS | Reduction in reliance on restraint techniques |
|  | Whittle et al., 2021 | PBS | The study shows a decrease in physical and restrictive interventions (approximately one-quarter from baseline) |
| b. Use of medication (other than PRN)  (n=2) | Whittle et al., 2021 | PBS | NR on medication use |
|  | Hackett et al., 2020 | Interpersonal art psychotherapy | NR on medication use |
| c. Incidents of ‘aggression’ or ‘behaviour that challenges’ (incl. self-harm)  (n=15) | Borghino et al., 2022 | Neurofeedback interventions | No incidents of accessing inappropriate materials or sexually inappropriate interactions were reported post-treatment |
|  | Alexander et al., 2011 | 10-point treatment programme | Transition to less restrictive environments indicating sufficient management of aggressive behaviour |
|  | Ashworth et al., 2021 | ‘I can feel good’ programme | Improved behavioural construct (impulsivity, emotional regulation) |
|  | Sakdalan & Collier, 2012 | SAFE-ID (Adapted DBT) | Reduction in physical, verbal aggression, and other problematic behaviours (including self-harm) six months after the group |
|  | Browne et al., 2019 | Intellectual disability-adapted dialectical behaviour therapy (DBT) skills group sessions | Reduction in aggression |
|  | Browning et al., 2016 | CFT | Decrease in severity of offense-related behaviours and high-risk behaviours like fire-setting and sexual offenses |
|  | Campbell & McCue, 2013 | AIR | Effective addition to managing interpersonal risk |
|  | Craven & Shelton, 2020 | Intellectual disability-adapted dialectical behaviour therapy (DBT) skills group sessions | Reduction in aggression and hostility was observed post-module, but not statistically significant |
|  | De Villiers & Johnstone, 2024 | PRISM protocol for violence reduction | Reduction in violent incidents |
|  | Hackett et al., 2020 | Interpersonal art psychotherapy | Frequency and severity of aggressive behaviour (measured by MOAS) reduced |
|  | Hickman et al., 2018b | Psychological treatment pathway | Reduction in aggressive and offending behaviour |
|  | Inett et al., 2014 | START and ISS tools | High predictive validity for risk and incidents of aggression |
|  | Langdon et al., 2013 | EQUIP | The study shows a significant decrease in scores related to physical aggression and self-harm on the HIT |
|  | Lindsay et al., 2013 | A comprehensive community service for offenders with ID | Reduction in number of incidents highly significant |
|  | Novaco & Taylor, 2015 | Individual-based CBT anger treatment | Decreased incidents of physical aggression, self-harm, and challenging behaviour |
|  | Singh et al., 2011 | Meditation on the Soles of the Feet for | Reduction in aggression |
|  | Taylor & Novaco, 2023 | Individual-based CBT anger treatment | Reduction in aggressive behaviour |
|  | Taylor et al., 2002a | Individual-based CBT anger treatment | Reduction in aggressive behaviour |
|  | Taylor et al., 2002b | A group intervention for convicted arsonists | Significant reduction in self-rated anger |
|  | Taylor et al., 2005 | Individual-based CBT anger treatment | Reduction in aggressive behaviour |
|  | Taylor et al., 2006 | Fire setters’ treatment programme | Reduction in anger, and depression post-treatment |
|  | Taylor et al., 2016 | Individual-based CBT anger treatment | Significant reduction in aggressive and violent incidents |
|  | Tearle et al., 2020 | EQUIP | Reduction in anger, and depression post-treatment |
|  | Travis & Sturmey, 2013 | BST | Decline in aggressive responses and improved replacement behaviours (non-aggressive verbal and physical responses) |
|  | Thomas et al., 2005 | MOACAP | A 79% reduction specifically in physical assaults. |
| **Patient/carers experience outcomes (n=13)** |  |  |  |
| a. Patient narratives of hospitalisation (if relevant to the intervention/care) (n=2) | Tearle et al., 2020 | EQUIP | Personal reflections on the emotional and practical aspects of discharge, offering insight into the lived experience of care and transition |
|  | Borghino et al., 2022 | Neurofeedback intervention | Qualitative feedback from John supported the positive impact of the intervention |
| b. Patient views of service/care/intervention (n=11) | Ashworth et al., 2020 | CBT for depression | Self-reported improvements in depression symptoms |
|  | Ashworth et al., 2017 | Mind matters | The programme was positively received by patients |
|  | Browne et al., 2019 | Intellectual disability-adapted dialectical behaviour therapy (DBT) skills group sessions | Participants shared what they found helpful or unhelpful about the adapted DBT programme |
|  | Borghino et al., 2022 | Neurofeedback intervention | Qualitative feedback from John supported the positive impact of the intervention |
|  | Hackett et al., 2020 | Interpersonal art psychotherapy | Patients reported high acceptability of the intervention through qualitative interviews |
|  | Hickman et al., 2018a | Leavers Preparation Group | Service users reported the group as positive and beneficial |
|  | Tearle et al., 2020 | EQUIP | Positive views of the programme |
|  | Morris et al., 2021 | Coproduction approaches to dynamic risk assessment (Short Dynamic Risk Scale) | Positive patient reflections on their participation in risk assessments and management planning. |
|  | Quinn et al., 2022 | Talking Mats | Patients found Talking Mats to be a helpful and non-biased platform for discussing sensitive topics like risk, safety, and wellbeing |
|  | Sakdalan & Collier, 2012 | (SOTSEC-ID) programme, individual and group-based | Positive feedback from patients of learning to effectively manage their behaviour |
|  | Singh et al., 2011 | Meditation on the Soles of the Feet, and a Mindful Observation of Thoughts meditation | Participants showed greater success with the mindfulness-based techniques than with their own self-control methods |
| c. Quality of Life (QoL) | None | None | None |
| d. Family/carers’ perspectives of care/treatment/intervention | None | None | None |
| e. Family contact/visits (n=1) | Cheshire et al., 2015 | Home visit programme within a forensic intellectual disability service | The audit found that 81% of patients had some form of family contact, and 54% had at least one home visit. |
| f. Met/unmet needs (n=2) | Ashworth et al., 2017 | Mind matters | The psychological and educational needs of individuals with intellectual disabilities and co-morbid mental disorders were met |
|  | Chaplin et al., 2024 | Specialist ND L&D service | The service identifies and support the needs of defendants with neurodevelopmental disorders, made reasonable adjustments and improve access to appropriate services |
| g. Complaints | None | None | None |
| h. CANDID-S | None | None | None |
| **Staff outcomes, skills and attributes (n=17)** |  |  |  |
| a. Stress/burnout (n=1) | Green & Cappleman, 2023 | RPG | RPGs helped the staff process emotional responses to demanding work |
| b. Staff turnover/sick leave | None | None | None |
| c. Skills/ training/ knowledge/ awareness/values/confidence/motivation/compassion (n=9) | Ashworth et al., 2017 | Mind matters | Benefited staff by reinforcing strategies for maintaining positive mental health |
|  | Ashworth et al., 2016 | Training programme for staff around the I Can Feel Good programme | Staff showed significant increases in self-reported knowledge, confidence, and motivation across seven training areas |
|  | Chaplin et al., 2024 | Specialist ND L&D service | The training evaluation reported knowledge sharing and awareness raising as key benefits |
|  | Chester et al., 2018 | Brain Injury Screening Index (BISI©) | Staff may benefit from improved awareness and training in TBI screening, but this is not formally evaluated. |
|  | Devapriam et al., 2014 | A core care pathway for referral, assessment, treatment and discharge | Improved service delivery through structured pathways |
|  | De Villiers & Johnstone, 2024 | PRISM protocol for violence reduction | PRISM protocol helped staff and management identify situational risk factors and implement changes |
|  | Morris et al., 2021 | Coproduction approaches to dynamic risk assessment (Short Dynamic Risk Scale) | Co-production improved engagement with service users |
|  | Taylor et al., 2003 | Introductory workshop designed for direct care staff working with sex offenders with ID | Qualified therapists were more effective in helping patients develop complex anger control coping skills |
|  | Whittle et al., 2021 | PBS | Staff reported increased knowledge, confidence, and sense of safety following training in proactive interventions and positive behaviour support |
| d. Experiences/views (n=10) | Ashworth et al., 2017 | Mind matters | Positive feedback from staff as beneficial for both patients and ward environment |
|  | Ashworth et al., 2016 | Training programme for staff around the I Can Feel Good programme | Staff feedback indicated a positive reaction to the training. |
|  | Chaplin et al., 2024 | Specialist ND L&D service | Semi-structured interviews reported their positive views on the service’s implementation and effectiveness |
|  | Green & Cappleman, 2023 | RPG | Staff perceptions of RPGs, including barriers to attendance and perceived value. |
|  | McKinnon et al., 2024 | W2W | W2W model was judged to be less embedded and sustained over time. |
|  | Quinn et al., 2022 | Talking Mats | Nurses appreciated Talking Mats as a tool that facilitated therapeutic conversations, especially around emotionally charged topics |
|  | Sakdalan & Collier, 2012 | (SOTSEC-ID) programme, individual and group-based | Positive feedback from staff as they observed improvement in patient behaviour. |
|  | Taylor et a., 2017 | Discharge pathway protocol | Stakeholders including staff reported the protocol as helpful in facilitating safe and effective discharges |
|  | Thomas et al., 2005 | MOACAP | Staff found the structured approach helpful in reducing aggressive incidents and improving consistency in care delivery |
|  | Wark & Gredecki, 2023 | CFS | Staff reported the proposed service delivery model as supportive of good practice |
| e. Quality of life (QoL) | None | None | None |

*Note.* n = number of studies
